# Tuning Immobilized Enzyme Features by Combining Solid-Phase Physicochemical Modification and Mineralization

**DOI:** 10.3390/ijms232112808

**Published:** 2022-10-24

**Authors:** José R. Guimarães, Diego Carballares, Javier Rocha-Martin, Paulo W. Tardioli, Roberto Fernandez-Lafuente

**Affiliations:** 1 Departamento de Biocatálisis, ICP-CSIC, Campus UAM-CSIC, 28049 Madrid, Spain; 2 Graduate Program in Chemical Engineering (PPGEQ), Laboratory of Enzyme Technologies (LabEnz), Department of Chemical Engineering, Federal University of São Carlos (DEQ/UFSCar), Rod. Washington Luís, km 235, São Carlos 13565-905, Brazil; 3 Department of Biochemistry and Molecular Biology, Faculty of Biology, Complutense University of Madrid, C/José Antonio Novais 12, 28040 Madrid, Spain; 4 Center of Excellence in Bionanoscience Research, Member of the External Scientific Advisory Board, King Abdulaziz University, Jeddah 21589, Saudi Arabia

**Keywords:** solid phase enzyme modification, immobilized lipase physicochemical modification, immobilized lipase mineralization, enzyme features tuning

## Abstract

Lipase B from *Candida antarctica* (CALB) and lipase from *Thermomyces lanuginosus* (TLL) were immobilized on octyl agarose. Then, the biocatalysts were chemically modified using glutaraldehyde, trinitrobenzenesulfonic acid or ethylenediamine and carbodiimide, or physically coated with ionic polymers, such as polyethylenimine (PEI) and dextran sulfate. These produced alterations of the enzyme activities have, in most cases, negative effects with some substrates and positive with other ones (e.g., amination of immobilized TLL increases the activity versus *p*-nitro phenyl butyrate (*p*-NPB), reduces the activity with *R*-methyl mandate by half and maintains the activity with *S*-isomer). The modification with PEI increased the biocatalyst activity 8-fold versus *R*-methyl mandelate. Enzyme stability was also modified, usually showing an improvement (e.g., the modification of immobilized TLL with PEI or glutaraldehyde enabled to maintain more than 70% of the initial activity, while the unmodified enzyme maintained less than 50%). The immobilized enzymes were also mineralized by using phosphate metals (Zn^2+^, Co^2+^, Cu^2+^, Ni^2+^ or Mg^2+^), and this affected also the enzyme activity, specificity (e.g., immobilized TLL increased its activity after zinc mineralization versus triacetin, while decreased its activity versus all the other assayed substrates) and stability (e.g., the same modification increase the residual stability from almost 0 to more than 60%). Depending on the enzyme, a metal could be positively, neutrally or negatively affected for a specific feature. Finally, we analyzed if the chemical modification could, somehow, tune the effects of the mineralization. Effectively, the same mineralization could have very different effects on the same immobilized enzyme if it was previously submitted to different physicochemical modifications. The same mineralization could present different effects on the enzyme activity, specificity or stability, depending on the previous modification performed on the enzyme, showing that these previous enzyme modifications alter the effects of the mineralization on enzyme features. For example, TLL modified with glutaraldehyde and treated with zinc salts increased its activity using *R*-methyl mandelate, while almost maintaining its activity versus the other unaltered substrates, whereas the aminated TLL maintained its activity with both methyl mandelate isomers, while it decreased with *p*-NPB and triacetin. TLL was found to be easier to tune than CALB by the strategies used in this paper. In this way, the combination of chemical or physical modifications of enzymes before their mineralization increases the range of modification of features that the immobilized enzyme can experienced, enabling to enlarge the biocatalyst library.

## 1. Introduction

Enzymes are attracting increasing attention as industrial catalysts due to their high activity under mild conditions, high substrate specificity and product selectivity [1,2,3], with the huge evolution of the strategies to solve their limitations imposed by their biological origin. Metagenomics enable to utilize the whole biodiversity (making enzymes from non-cultivable or even disappeared organisms available) [4,5,6], genetic tools enable to tailor the desired feature (using enzyme modeling and site-directed mutagenesis [7,8] or directed evolution [9,10,11]), and physicochemical enzyme modifications are more efficient and controlled each passing day. This enables a battery of solutions to overcome any enzyme deficiency [12,13]. Moreover, some of these solutions may be used in a conjoined way, for example, making the building of enzymes bearing several active centers feasible [14], such as plurizymes [15,16,17].

In this context, enzyme immobilization may be remarked upon. It was initially developed as a solution to the problems raised by enzyme solubility; the preparation of heterogeneous biocatalysts made enzyme recovery and reuse simpler (if the enzyme was still active) [18,19,20]. Nowadays, many researchers have shown that immobilization may solve many enzyme limitations. One of the most pursued objectives is enzyme stabilization, which may be accomplished by diverse reasons [21,22]. The fact that the enzyme is distorted when immobilized and is in a confined space (using porous supports) also produces an alteration of enzyme selectivity, specificity, activity, response to inhibitors, etc. [23,24,25], that can greatly increase the reactions where the enzyme can be utilized with satisfactory results. Moreover, the enzyme may be purified during the immobilization process [26,27].

An especially interesting possibility is the simultaneous use of immobilization as a way to simplify other enzyme improvement strategies. That way, the solid-phase chemical modification of enzymes is much simpler than the modification of the free enzyme. It also prevents undesired enzyme aggregations, and if the enzyme is stabilized by the immobilization, it may be also more resistant to changes in the physical features of the enzyme surface caused by the chemical modification [28,29,30,31]. Recently, it was proposed also that the mineralization of enzymes in the solid phase may have some advantages [32,33]. The building of enzyme hybrid nanoflowers (where a salt crystal grows around the enzyme molecules that act as nucleation points) as an immobilization system has proved to allow the improvement of enzyme stability and activity in certain cases [34,35,36,37,38,39,40,41,42,43], but the small size and fragility of the resulting structures makes their recovery complex. Some researchers have proposed the use of magnetic materials or the trapping of these nanoflowers in larger and mechanically more stable structures as a solution for these limitations [44,45,46,47,48,49,50,51]. The mineralization of previously immobilized lipases was recently proposed as a possibility to obtain some benefits from mineralization but with a mechanically stable biocatalyst [32,33]. It has been shown that the use of fully loaded biocatalyst permits to maximize the effects, and that the phosphate metal crystals also grow in the support pores even in absence of enzymes with similar intensity [32]. Moreover, it has been shown that the enzyme orientation and/or enzyme conformation can greatly alter the final effects of this mineralization on enzyme features [52]. In this new communication, we evaluate if the enzyme chemical modification of immobilized enzymes can somehow alter the results observed by the enzyme mineralization [52]. It has been shown in many papers how the chemical or physical modification of immobilized enzymes may have different effects depending on the enzyme immobilization protocol [31,53,54,55,56]. The enzyme mineralization is really a physical modification of the enzyme, and it can be expected that the effects may depend on the enzyme exact conformation and orientation concerning the support. In fact, it has been shown that some ions may be very negative or positive for immobilized lipase stability, but only when the enzymes are immobilized following certain specific protocols [57,58].

This may depend on the exact chemical composition of the protein external surface that will be the subject of the modification. Different chemical compositions may lead to different degrees of enzyme coating by the metal crystals. In fact, recently, researchers have shown how protein folding and molecular modification define the rate, extent, and mechanism of crystallization when mixed with 2-methylimidazole and zinc acetate [59]. We can assume that these effects may be also produced when mineralizing immobilized enzymes, and that they can produce different effects on the enzyme properties.

Lipases are used as model enzymes in this study, as they are among the most used enzymes at both academic and industrial levels [60,61,62,63]. They have a peculiar mechanism of action, called interfacial activation, as they have two possible conformations (closed and open), that in homogeneous media are in equilibrium [64,65,66,67]. A polypeptide called lid is able to isolate the active center in most lipases. In the presence of any hydrophobic surface (a drop of substrate, a hydrophobic protein, but also other open form of the lipase or a hydrophobic support), the lipase becomes strongly adsorbed, with the lid shift leaving the active center exposed [64,65,66,67]. This has caused lipase immobilization on hydrophobic supports to become a very popular immobilization strategy, enabling the one-step immobilization, purification, stabilization and hyperactivation of the lipases [27]. This is the immobilization strategy utilized in this paper, using octyl-agarose beads as support [68]. Then, the immobilized enzymes are submitted to diverse physicochemical modifications to analyze if this can tune the effects of the mineralization. Moreover, we can observe if some of the modified groups are necessary to obtain the mineralization effects, or not. The first modification is that using glutaraldehyde [69,70]. The utilized modification conditions with glutaraldehyde ensures the full modification of all primary amino groups in the enzyme surface with just one molecule of glutaraldehyde [71], making the enzyme surface slightly more hydrophobic (glutaraldehyde is mildly hydrophobic) but leaving the ionization capability to the amino group. This can stabilize the enzyme due to the promotion of inter-(making enzyme release more difficult) [72] and intramolecular (increasing enzyme rigidity) crosslinkings [73]. The amination of the enzyme with ethylenediamine (EDA) and carbodiimide permits to transform all external carboxylic groups in amino groups, altering the ionic interactions (now, all will be repulsion interactions) [31,74,75]. Finally, the modification of the enzyme with picrylsulfonic acid or trinitrobenzenesulfonic acid (TNBS) is highly selective for the primary amino groups [76], and makes the ionization capability of the amino group disappear, as it becomes an amide, promoting enzyme surface hydrophobization. Finally, the enzyme surface is coated using polyethyleneimine (PEI) [77], or dextran sulfate [78,79]; both polymers will almost fully coat the enzyme surface and permit the intermolecular crosslinking, making enzyme release from the support difficult [80]. The polymers offer an open structure, permitting the entry of small compounds to the enzyme surface, but generating some partition effects due to their ionic character. In this study, we selected the lipases from *Thermomyces lanuginosus* [81] and *Candida antarctica* (form B) [82,83,84], which are among the most popular ones; the solid phase mineralization [32,33] and the chemical modification presented interesting results in previous publications using both enzymes [55,56,72,85,86,87,88,89]. That way, the objectives of this paper can be summarized in an effort to analyze whether the coupled solid phase chemical or physical modification of enzymes and their further mineralization may have additive effects on the enzyme features, the results from the first strategy influencing the results achieved by the second one.

## 2. Results and Discussion

### 2.1. Preparation of the Immobilized and Chemically Modified TLL-Biocatalysts

TLL was immobilized on octyl agarose, as shown in Figure S1; around 50% of the enzyme activity was incorporated to the support after 2 h (time found in previous papers to be enough to fully coat the support surface with lipases under these conditions) [90,91,92,93,94,95]. Then, the biocatalyst was submitted to the different modifications described in introduction. Table 1 summarizes the effect of the modification on the activity versus different substrates. The highest activity was found using the *p*-NPB assay, and the lowest one using the *R*-methyl mandelate, being that the activity was more than 2.5-fold higher using the *S*-isomer. The modification with glutaraldehyde marginally decreased the activity using the *p*-NPB assay, decreased its activity by 20% in the triacetin assay, decreased by 1/3 using the *R*-methyl mandelate, and decreased by 1/4 using the *S*-methyl mandelate assays (increasing the activities ratio). The amination produced almost a 20% increase in the activity versus *p*-NPB, and a 44% increase versus triacetin, while the activity versus *R*-methyl mandelate decreased to 50%, and that versus the *S*-isomer was maintained (doubling the activities ratio). The modification with TNBS increased the activity using *p*-NPB by 25%. The activity was not affected when using triacetin, and it decreased using the *R*-methyl mandelate, while it almost did not affect the activity using the *S*-isomer (again increase the activities ratio). That way, all these chemical modifications affected enzyme specificity and enantiospecificity, in many cases based on an increase in the activity using some of the activity assays. To explain the reasons for these effects is complex, as it has been shown that the effect of the chemical or physical modification not only depends on the enzyme, but also on the immobilization protocol [31,53,54,55,56].

The physical coating of the immobilized TLL with PEI produced a small decrease in the activity using the *p*-NPB assay, but the activity almost increased by 50% using triacetin. The most interesting result is the great increase in the immobilized enzyme activity versus both methyl mandelates: 8.2-fold using the *R*-isomer, and 2.4-fold using the *S*-isomer, with a preference for the *R*-isomer from the enzyme. The modification using DS decreased the activity using *p*-NPB (to 70%) and triacetin (to 46%), while it significantly increased the activity versus the methyl mandelates by 6.25 using the *R*-isomer and by just 2-fold using the *S*-isomer. Again, the modified enzyme prefers the *R*-isomer. That way, these modifications also strongly tuned the enzyme specificity, as previously described [55,56,72,85,86,87,88,89].

**Figure 1:**
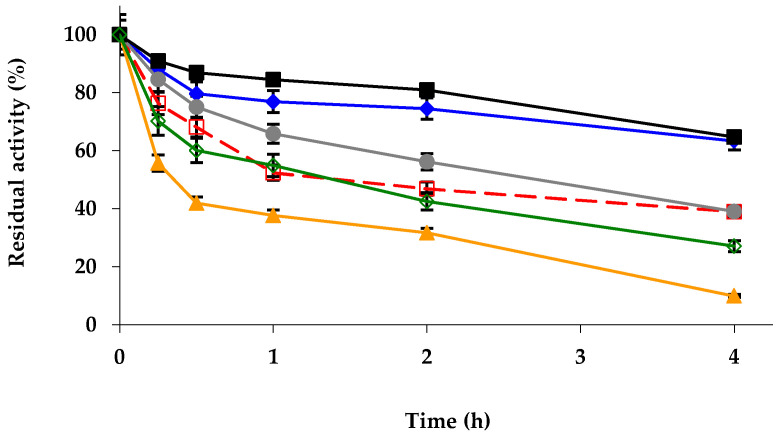
Inactivation courses of different physically and chemically modified octyl-TLL biocatalysts. The biocatalysts were inactivated at 68 °C, in presence of 10 mM Tris-HCl buffer at pH 7.0. Other specifications are described in Methods. Unmodified octyl-TLL (empty squares and dashed and red line); octyl-TLL modified with 1% glutaraldehyde (solid rhombus and solid blue line); amination using 2 M ethylenediamine (solid triangles and solid orange line); 1 mM picrylsulfonic acid (solid circles and solid grey line); 10% polyethylenimine (solid squares and solid black line); 10% dextran sulfate (empty rhombus and solid green line).

Next, the stabilities of the different biocatalysts were evaluated (Figure 1). The modifications with glutaraldehyde and PEI produced significant stabilizations, while the modification with TNBS and DS almost did not affect enzyme stability, and the enzyme amination promoted a decrease in enzyme stability. It should be considered that the proximity of the enzyme molecules (we are using fully loaded biocatalysts) can affect enzyme stability, and that the chemical modifications will not only affect the intramolecular enzyme interactions or the possibility of intermolecular crosslinking, but also the enzyme–molecule/enzyme–molecule interactions may be altered [90,91,96]. In any case, it seems that the inter- and intramolecular crosslinkings induced by glutaraldehyde can have positive effects on the immobilized enzyme stability (rigidifying the enzyme structure and hindering enzyme desorption from the support) [70,72,97] or the intermolecular crosslinking achieved by using PEI for the enzyme stability [78,80]. DS should produce similar crosslinking, but apparently the anionic environment generated by this molecule is not positive for enzyme stability.

### 2.2. Modification of Octyl-TLL with Different Phosphate Salts

Next, the effects of the modification of octyl-TLL biocatalyst with Zn^2+^, Co^2+^, Cu^2+^, Ni^2+^ and Mg^2+^ on its activity and stability were studied. Table 2 summarizes the activity results. In the *p*-NPB assay, only the modification with Mg^2+^ produced a slight increase in enzyme activity (by more than 15%), being the less active biocatalysts that modified using Cu^2+^ (with an activity just over 80%). In the case of the triacetin assay, the activity only decreased using Ni^2+^ (to around 88%), while the other modifications slightly increased the activity in this assay by around 10%. Using *R*-methyl mandelate, Ni^2+^ and Mg^2+^ produced an increase in the enzyme activity (by 25%), and the Cu^2+^ treatment produced a drastic decrease in activity (by 1/3), while Zn^2+^ had a marginal effect. Using the *S*-isomer, all biocatalysts decreased their activity, being that the enzyme treated with Mg^2+^ is the least active one (75% activity compared to the untreated biocatalyst). That way, the *R/S* activities ratio changed after the different mineralizations. This shows how this mineralization is also able to tune the immobilized enzyme specificity; it is not possible to state a mineralization as being universally positive or negative for octyl-TLL activity, as that depends on the substrate. These effects should be the results of conformational changes induced by the mineralization; these changes yield a more active enzyme form for some substrates, while for other substrates, the effects are negative.

**Figure 2:**
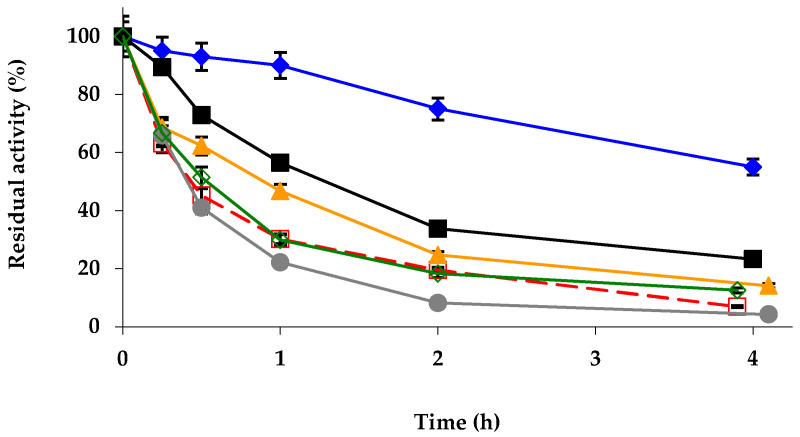
Inactivation courses of different octyl-TLL biocatalysts in 10 mM Tris-HCl buffer at pH 7.0 and 75 °C. Other specifications are described in Methods. Unmodified octyl-TLL (empty squares and dashed and red line); octyl-TLL modified with 1% glutaraldehyde (solid rhombus and solid blue line); amination using 2 M ethylenediamine (solid triangles and solid orange line); 1 mM picrylsulfonic acid (solid circles and solid grey line); 10% polyethylenimine (solid squares and solid black line); 10% dextran sulfate (empty rhombus and solid green line).

The stabilities of the different biocatalysts were then analyzed (Figure 2): the mineralization with Zn^2+^ produced the highest stabilization; Ni^2+^ produced also a relevant stabilization; the Co^2+^ treatment produced a lower stabilization; and the Cu^2+^ treatment promoted a slight destabilization. Mg^2+^ mineralization had no effect on enzyme stability.

Next, we have compared these mineralization effects using the enzymes previously modified.

### 2.3. Effect of Mineralization on the Immobilized Enzyme Previously Modified

Starting with the enzyme modified with glutaraldehyde, Figure S2 shows that the color acquired by the glutaraldehyde modified biocatalysts was fairly similar to that observed using the unmodified biocatalyst for all metals. The mineralization produced a slight increase in *p*-NPB activity using Zn^2+^ and Mg^2+^, and Ni^2+^ has almost no effect, while Co^2+^ and Cu^2+^ produced a decrease by over 15% (Table 3, Line 2–7). Using triacetin, the activity was almost unaltered using Zn^2+^, Mg^2+^ and Ni^2+^, while it decreased using Co^2+^ and Cu^2+^ (by almost 30%). All mineralization produced a decrease in the activity versus *R*-methyl mandelate, which was not very relevant except when using Co^2+^ (the activity becomes 25%); using the *S*-isomer, the picture is quite different, as the Zn^2+^ treatment increased the activity by 25%, Mg^2+^ maintained the activity, and Ni^2+^, Cu^2+^ and Co^2+^ produced the highest decrease in activity (from 2.4 to 2.2, 2.1 and 2.0, respectively) (Table 3, Line 2–7). That way, enzyme activities and specificity were altered, although in a different form to the mineralization of the unmodified biocatalyst. The changes in activity should be related to alterations in the enzymes conformation, which are positive for some substrates and negative for others.

**Figure 3:**
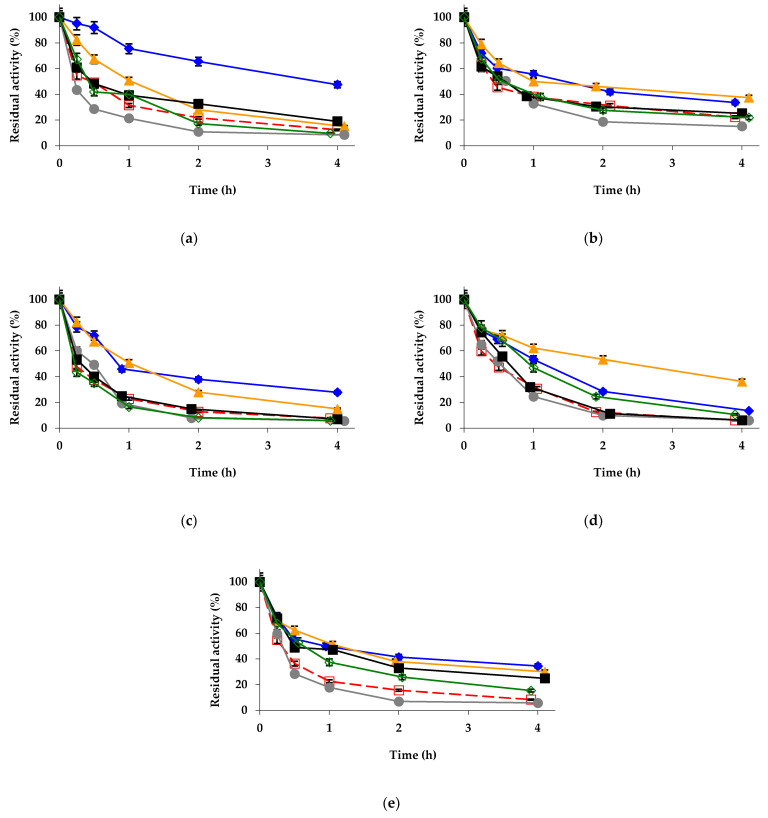
Inactivation courses of different octyl-TLL biocatalysts modified with (**a**) 1% glutaraldehyde, (**b**) amination using 2 M ethylenediamine, (**c**) 1 mM picrylsulfonic acid, (**d**) 10% polyethyleneimine, and (**e**) 10% dextran sulfate. The biocatalysts were inactivated at 68 °C (**b**) and 75 °C (**a**,**c**–**e**), in presence of 10 mM Tris-HCl buffer at pH 7.0. Other specifications are described in Methods. Unmodified octyl-TLL (empty squares and dashed and red line); octyl-TLL modified with 1% glutaraldehyde (solid rhombus and solid blue line); amination using 2 M ethylenediamine (solid triangles and solid orange line); 1 mM picrylsulfonic acid (solid circles and solid grey line); 10% polyethylenimine (solid squares and solid black line); 10% dextran sulfate (empty rhombus and solid green line).

Regarding the effect on the enzyme stability, the stabilizing effect of glutaraldehyde made it necessary to increase the temperature to 75 °C to visualize the inactivation of the different biocatalysts in a reasonable timeframe (Figure 3a). Zn^2+^ treatment maintained its great stabilization effect, Co^2+^ and Ni^2+^ produced a slight stabilization, Mg^2+^ mineralization almost had no effect, while copper produced a slight destabilization. In this regard, the situation was not very different from the effects detected using the unmodified biocatalyst. Considering the previous stabilization achieved using glutaraldehyde, the additive effect of the zinc treatment produced a highly stabilized biocatalyst (Figure 3a). The glutaraldehyde + Zn^2 +^-treated biocatalyst maintained levels of residual activity similar to the those of the only-mineralized biocatalysts after 4 h but at 7 °C higher temperature (Figure 1 and Figure 2). It seems that the positive effect from glutaraldehyde on enzyme stability was additive to those of zinc mineralization.

The mineralization of the aminated biocatalyst gave similar colors to those of the unmodified biocatalysts (Figure S2). It produced also significant changes to enzyme specificity (Table 3, Line 8–13). The activity using *p*-NPB assay was not too altered, producing a slight decrease on the enzyme activity in most cases (by a maximum of 15% using Cu^2+^), except for a very slight increase using Ni^2+^ (by less than 5%). Using triacetin as a substrate, again, all mineralized biocatalysts decreased their activity (with the most deleterious modifications being those performed using with cobalt, which decreased the activity from 88 U/g to 5.3 U/g). Using the *R*-methyl mandelate, the enzyme activity remained almost unaltered when mineralized with Ni^2+^ and Zn^2+^, while drastically decreasing using Co^2+^ (from 0.6 to 0.1 U/g) and increasing when the modification was performed using Cu^2+^ (by 50%) or Mg^2+^ (by 35%). Using the *S*-isomer, all mineralized biocatalysts decreased their activity slightly, except when using Co^2+^, which produced a decrease in the enzyme activity from 3.2 to 2.2. These activity changes were translated to significant changes on enzyme specificity and enantiospecificity (Table 3, Line 8–13). Regarding the effects on enzyme stability (inactivation was at 68 °C, as amination was negative for enzyme stability), Figure 3b shows that Zn^2+^ and Co^2+^ mineralization produced a small stabilization of the biocatalyst, while treatment with Mg^2+^ and Ni^2+^ left the stability unaltered and Cu^2+^ produced a small destabilization. The effects were much smaller than those found using the unmodified immobilized enzyme (Figure 2).

The mineralization of the enzyme modified with TNBS gives a different color from the initially exhibited by the biocatalysts (Figure S2), as these preparations presented a yellow-orange color. The treatment produced a slight decrease in *p*-NPB activity (minimum value was observed for the Cu^2+^ modification, decreasing the activity by 17%) (Table 3, Line 14–19). The activity versus triacetin was increased in all cases, with the maximum value being that observed using Cu^2+^ mineralization (by almost 1.9) and the minimum one that obtained using Zn^2+^ (by around 1.5). The activities versus both isomers of methyl mandelate were fairly maintained (Table 3, Line 14–19). Regarding the stability (Figure 3c), it was increased using Zn^2+^ and Co^2+^, while the other mineralizations produced marginal effects.

The biocatalysts coated with PEI presented more intense colors than the unmodified biocatalyst, suggesting that PEI can capture some metal salts (Figure S2). These modified biocatalysts slightly increased the activity versus *p*-NPB when using Co^2+^, Cu^2+^ or Zn^2+^ (this last gave the highest value, increasing the activity by more than 15%), while Ni^2+^ and Mg^2+^ treatment slightly decreased the activity (by around 10%) (Table 4, Line 2–7). Using triacetin, again, all biocatalysts increased their activity, and again Cu^2+^ mineralization gave the highest value (increasing the activity by less than 55%). Using methyl mandelate isomers, the results were quite different, using the *R*-isomer, while Zn^2+^ and Co^2+^ almost did not affect enzyme activity, Mg^2+^, Cu^2+^ and mainly Ni^2+^ treatments decreased the activity (to less than 50% in the last case), while using the *S*-isomer, all biocatalysts increased their activity, with the highest increases in the immobilized enzyme activity being those observed using Ni^2+^ and Mg^2+^ mineralization (almost by 50%). Again, the changes in enzyme activity and specificity were quite relevant (Table 4, Line 2–7). Figure 3d shows the inactivation courses of these biocatalysts. In this instance, the highest stabilization was found using Co^2+^ treatment, with the Mg^2+^ and Zn^2+^ treatment being slightly positive, while Cu^2+^ and Ni^2+^ had no clear effects (Figure 3d). These results were quite different from those obtained using the unmodified immobilized enzyme (Figure 2).

Last, the enzyme coated with DS was also mineralized, with a color intensity similar to that of the unmodified biocatalyst (Figure S2). The effects on *p*-NPB activity (Table 4, Line 8–13) were negligible. We can remark on the decrease observed using Cu^2+^ (by less than 10%). Using triacetin, a general increase in the activity was detected, with maximum values using Mg^2+^ and Cu^2+^ (increased reaching 1.5 folds). The activities using the methyl mandelate were almost unaffected (a 15% increase for the *R*-isomer using Zn^2+^ and less than a 10% increase for the *S*-isomer using Cu^2+^) (Table 4, Line 8–13). Figure 3e shows the inactivation courses; most mineralization produced similar stabilization (not very high), with the lowest stabilization being that found when using Mg^2+^, except when employing Cu^2+^, which slightly reduced the enzyme stability.

That way, the results show that using octyl-TLL, the combination of chemical modification and mineralization may greatly alter the enzyme features (activity, specificity and stability). All biocatalysts maintained over 90% of their initial activity after incubation at pH 7 and 37 °C for one month (results not shown); that way, even the least stable ones could be utilized for many applications, thanks to the initial high stability of the enzyme immobilized on octyl-agarose. The color remained attached to the support.

### 2.4. Preparation of the Immobilized and Chemically Modified CALB-Biocatalysts

Figure S3 shows that only 50% of the enzyme was immobilized on the support, confirming that the loading capacity of the support was exceeded and that way, the full support surface will be coated with enzyme molecules.

Next, octyl-CALB was submitted to different chemical and physical modifications. First, the effect of these modifications on the enzyme activities was analyzed (Table 5). The modification with glutaraldehyde produced a slight increase in the *p*-NPB activity (by 15%), while the activity versus triacetin decreased almost by 30%, the activity versus *R*-methyl mandelate decreased by 15% and that versus the *S*-isomer by 30%. The amination of the enzyme produced a similar increase for the *p*-NPB assay, while with that versus triacetin, the decrease in activity was over 40%, the activity versus *R*-methyl mandelate increased by 35%, and that versus the *S*-isomer decreased by almost 30%. The TNBS modification produced again a 15% increase in the *p*-NPB activity; versus triacetin, the activity decreased by almost 35%; and versus the methyl mandelate esters, it remained almost unaltered. The coating with PEI produced a more significant increase in the *p*-NPB activity of the biocatalyst (by more than 30%), a small increase on the activity employing triacetin (by less than 10%), a 33% increase using *R*-methyl mandelate and a 15% decrease using the *S*-isomer (Table 5). That way, although not with the same intensity as using TLL (Table 1), significant changes in enzyme activity, specificity and enantiospecificity could be observed after the chemical modification of octyl-CALB.

**Figure 4:**
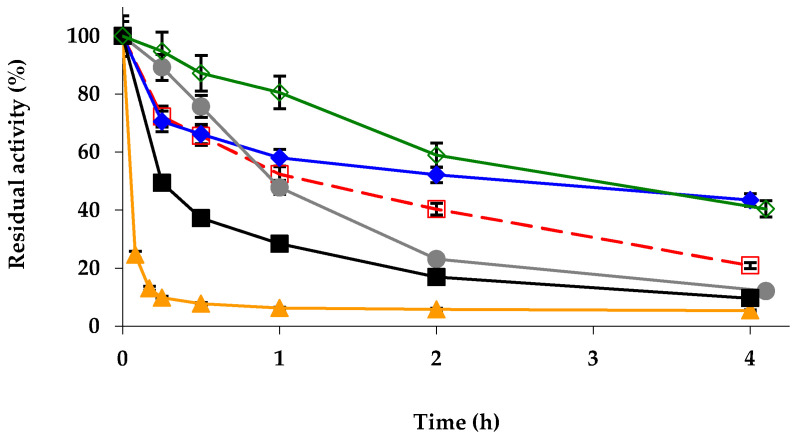
Inactivation courses of different physically and chemically modified octyl-CALB biocatalysts. The biocatalysts were inactivated at 75 °C in presence of 10 mM Tris-HCl buffer at pH 7.0. Other specifications are described in Methods. Unmodified octyl-TLL (empty squares and dashed and red line); octyl-TLL modified with 1% glutaraldehyde (solid rhombus and solid blue line); amination using 2 M ethylenediamine (solid triangles and solid orange line); 1 mM picrylsulfonic acid (solid circles and solid grey line); 10% polyethylenimine (solid squares and solid black line); 10% dextran sulfate (empty rhombus and solid green line).

Next, the effect of these modifications on the enzyme stability was analyzed (Figure 4). Glutaraldehyde and DS modifications permitted to increase enzyme stability, TNBS permitted an initial slowing of the inactivation but later it was faster than that of the unmodified enzyme, while PEI and mainly EDA modifications were to be negative for the enzyme stability. These results are very different from those found using TLL (Figure 1).

### 2.5. Mineralization of Octyl-CALB

**Figure 5:**
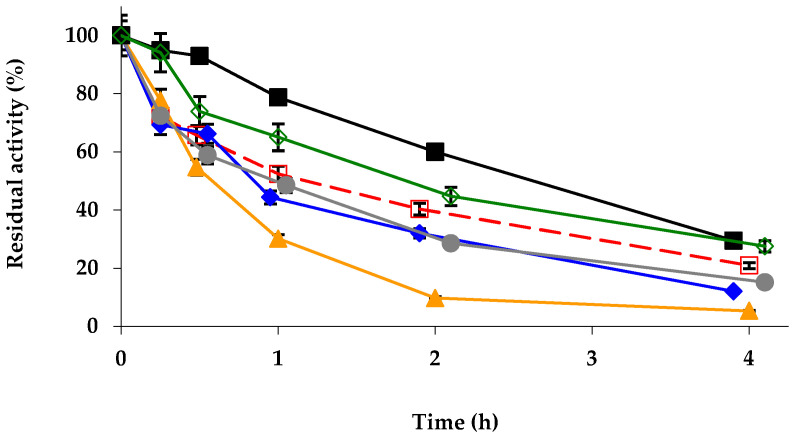
Inactivation courses of different octyl-CALB biocatalysts in 10 mM Tris-HCl buffer at pH 7.0 and 75 °C. Other specifications are described in Methods. Unmodified octyl-TLL (empty squares and dashed and red line); octyl-TLL modified with 1% glutaraldehyde (solid rhombus and solid blue line); amination using 2 M ethylenediamine (solid triangles and solid orange line); 1 mM picrylsulfonic acid (solid circles and solid grey line); 10% polyethylenimine (solid squares and solid black line); 10% dextran sulfate (empty rhombus and solid green line).

The effect of the enzyme mineralization with different metal phosphates on octyl-CALB activity versus different substrates may be found in Table 6. Zn^2+^ treatment maintained the activity versus *p*-NPB, increasing the activity versus triacetin (by around 45%) and both isomers of methyl mandelate (by around 30%). That meant that although enantiospecificity was almost unaltered, the enzyme activity and substrate specificity were quite altered. Regarding the effects on enzyme stability, Figure 5 shows that Mg^2+^ and more clearly Ni^2+^ metallization produced some enzyme stabilization, while Zn^2+^ and Cu^2+^ had a small negative effect, which was clearer using Co^2+^. These results are quite different from those found with TLL (Figure 2).

That way, even with the lower intensity compared to the case of TLL, the chemical modification and the mineralization of octyl-CALB produced diverse effects on enzyme specificity, activity and stability. Next, we analyzed if the chemical modification can somehow alter the effects of the mineralization on enzyme features, such as occurred using octyl-TLL.

### 2.6. Effect of Mineralization on the Immobilized Enzyme Previously Modified

The mineralization of all preparations gave similar colored biocatalysts (Figure S4); remarkably, the octyl-CALB-PEI presented a more intense color, suggesting a higher metal retention caused to the PEI. The exception is the case of TNBS, as the initial yellow-orange color alters the observed colors after mineralization.

Starting with the enzyme modified with glutaraldehyde, the effect on the *p*-NPB activity was not very significant (Table 7, Line 2–7); using Co^2+^, the activity was maintained; and using Ni^2+^, a small decrease could be detected (under 5%), the enzyme activity decrease being slightly higher than when using the other metals in the enzyme mineralization (9–12%). The activity versus triacetin slightly decreased for all treatments, the decrease of activity using Ni^2+^ being the most significant one (around 17%). Using the *R*-methyl mandelate, the decrease in activity was more significant in some instances; the activity became around 55% for the Zn^2+^ treated biocatalyst, and increased more than 10% using the Ni^2+^ metalized biocatalyst. The other biocatalysts almost did not alter its activity in this assay. Using the *S*-isomer, the Zn^2+^ treated biocatalyst remained the least active biocatalyst, with less than 11% decrease in the enzyme activity. Cu^2+^ treatment increased the activity in a similar way, and the other biocatalysts were in between. This way, the glutaraldehyde modification decreased the effects of the mineralization on the immobilized CALB activity.

**Figure 6:**
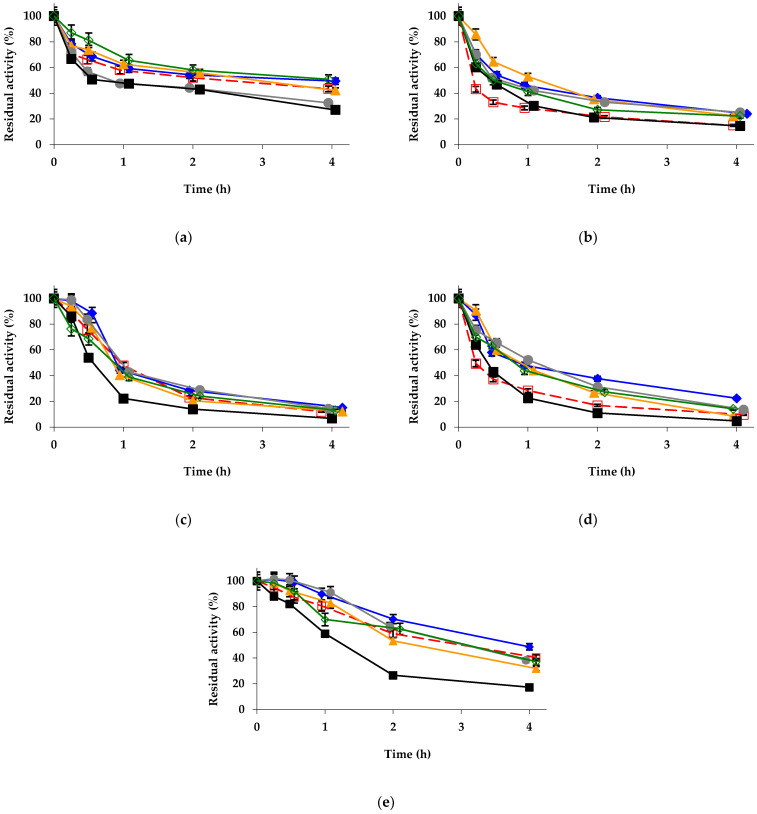
Inactivation courses of different octyl-CALB biocatalysts modified with (**a**) 1% glutaraldehyde, (**b**) amination using 2 M ethylenediamine, (**c**) 1 mM picrylsulfonic acid, (**d**) 10% polyethylenimine, and (**e**) 10% dextran sulfate. The biocatalysts were inactivated at 68 °C (**b**) and 75 °C (**a**,**c**–**e**), in presence of 10 mM Tris-HCl buffer at pH 7.0. Other specifications are described in Methods. Unmodified octyl-TLL (empty squares and dashed and red line); octyl-TLL modified with 1% glutaraldehyde (solid rhombus and solid blue line); amination using 2 M ethylenediamine (solid triangles and solid orange line); 1 mM picrylsulfonic acid (solid circles and solid grey line); 10% polyethylenimine (solid squares and solid black line); 10% dextran sulfate (empty rhombus and solid green line).

Analyzing the effects of the mineralization on the octyl-CALB-GA on enzyme stability (Figure 6a), it is easy to visualize that the mineralization effects were also decreased. Zn^2+^, Co^2+^ and Mg^2+^ presented a negligible positive effect on enzyme stability, while Cu^2+^ and Ni^2+^ produced a slight destabilization, clearer than the stabilization effects of the other metals. The results disagreed with those observed using the unmodified biocatalysts, both in intensity and in quality (e.g., Co^2+^ effects).

Going to the aminated biocatalyst, the effects on the *p*-NPB activity of the mineralization was reduced (Table 7, Line 8–13), becoming slightly positive for Ni^2+^ and Cu^2+^ treatments and slightly negative for the other metals (never exceeding 10% variations). Using triacetin, the mineralization produced a general increase in activity. The highest one was that obtained using Co^2+^ and Mg^2+^ (by more than 55%), and the lowest one was that observed after treatment using Cu^2+^ (by around 25%). When using the *R*-methyl mandelate, the activities slightly decreased, with minimum values using Zn^2+^ and Mg^2+^ (decreasing the activity to 90–89%). Using the *S*-isomer, only the Ni^2+^ treatment increased the activity very slightly (by less than 5%), while the other metals produced a relevant decrease in enzyme activity (to a minimum of 75%) (Table 7, Line 8–13). These effects were quite different to those found using glutaraldehyde modified preparations or the unmodified biocatalyst. Figure 6b shows that all mineralizations improved the enzyme stability (not in a very significant way). Co^2+^, Zn^2+^ and Cu^2+^ treatments showed the best stabilization effects.

The mineralization of the TNBS-modified CALB biocatalyst produced a slight decrease in *p*-NPB activity (by 13% in the highest case, using Mg^2+^) (Table 7, Line 14–19) but relevantly increased the activity versus triacetin in all cases, being the treatment with the highest increase in immobilized enzyme activity the one obtained using Cu^2+^ (1.9-fold), and the lowest being that obtained using Zn^2+^ and Co^2+^ (more than 1.5-fold). Using both isomers of methyl mandelate, the changes never exceeded 10%. The effects on enzyme stability may be visualized on the inactivation courses represented in Figure 6c; only Ni^2+^ treated biocatalyst slightly decreased its stability, and all the others biocatalysts had no significant differences.

Using the PEI-coated biocatalyst, the enzyme activity versus *p*-NPB increased using Zn^2+^ (by more than 15%) (Table 8, Line 2–7). As this was additive to the increase in immobilized enzyme activity obtained by the PEI coating, this converted this biocatalyst to being the most active versus this substrate. Additionally, Co^2+^ and Cu^2+^ mineralization increased the enzyme activity (by 7–8%). However, the modifications with Ni^2+^ and Mg^2+^ had no effects. Using triacetin, all mineralizations improved the enzyme activity, by around 50%, except using Zn^2+^ (this gave an activity of 125%). The activity versus *R*-methyl mandelate decreased using Mg^2+^ (to 73%), Cu^2+^ (to 66%) and Ni^2+^ (to 48%), while the other two metals had no relevant effects. In the activity detected using the *S*-isomer, all biocatalysts increased their activities, those treated with Cu^2+^ and Mg^2+^ being the most active (increasing the activity by 45%) (Table 8, Line 2–7). This promoted large changes in enzyme specificity, activity and enantiospecificity, different to those detected mineralizing the unmodified biocatalyst (Table 5). Regarding the effects on enzyme stability, Figure 6d shows that the mineralization with Ni^2+^ presented a slightly negative effect, while all the others slightly improved enzyme stability (increasing the half-life by 2–4-fold).

Using DS-coated biocatalyst, the enzyme *p*-NPB activity did not change in a relevant way, except for the Cu^2+^ treated biocatalyst, which lost almost 9% of its activity (Table 8, Line 8–13). The activity using triacetin increased in all cases, with the highest increase in immobilized enzyme activity around 50% using Cu^2+^ and Mg^2+^. The activity versus *R*-methyl mandelate was maintained or even increased (by 18% using Zn^2+^), in a similar trend to the unmodified biocatalyst and differently from the other modified biocatalyst. Using the *S*-isomer, its activity was maintained or slightly increased (by a maximum of only 6%). This was different from the unmodified biocatalyst, where the activity increased in a more significant way and for more metalizations (see Table 6). Enzyme stability was significantly decreased using Ni^2+^ treatment, while the treatments with the other metals had scarce effects (Figure 6e).

## 3. Materials and Methods

### 3.1. Materials

A TLL liquid formulation with 20.77 mg protein/mL was utilized in this paper, while lipase B from *Candida antarctica* (CALB) was a liquid formulation with 7.7 mg protein (kindly donated by Novozymes Spain (Madrid, Spain). Bradford’s reagent (utilized to calculate the protein concentration [98]), *p*-nitrophenyl-butyrate (*p*-NPB), triacetin, *R*- and *S*-methyl mandelate, acetonitrile for HPLC (gradient grade, ≥99.9%), glutaraldehyde (GA) solution (25% in H_2_O), ethylenediamine (EDA), picrylsulfonic acid (TNBS), polyethylenimine (PEI, MW 25,000), dextran sulfate (DS, MW 20,000), N-3-(dimethylaminopropyl)-N-ethylcarbodiimide hydrochloride (ECD), NiCl_2_, MgCl_2_, CoCl_2_, CuCl_2_ and ZnCl_2_ were purchased from Sigma-Aldrich (St. Louis, MO, USA). Octyl Sepharose^®^ CL-4B was acquired from GE Healthcare (Uppsala, Sweden). All other reagents were of analytical grade.

### 3.2. Methods

All experiments were performed at least in triplicate, and the values are presented as mean values and standard deviation.

#### 3.2.1. Immobilization of Lipases on Octyl-Agarose Beads

The lipases were immobilized by interfacial activation on octyl agarose beads using enzyme loads over the capacity of the support to ensure the full support of the surface coating (TLL: 20 mg/g and CALB: 25 mg/g) [99,100]. An amount of 1 g of support was added to 10 mL of the enzyme solution prepared in 5 mM sodium phosphate at pH 7.0. The immobilization was conducted at room temperature under gentle stirring for 2 h. The enzyme activity in the supernatant, suspension and a reference were quantified using *p*-NPB assay throughout the immobilization course. Afterward, the suspensions were vacuum filtered, washed 10-fold with 20 volumes of distilled water, and stored at 4–6 °C.

#### 3.2.2. Immobilization of Lipases on Octyl-Agarose Beads

The immobilized enzymes were treated with 1% (*v*/*v*) GA, aminated using 2 M of EDA following the carbodiimide route, or modified with 1 mM TNBS, 10% (*w*/*v*), 10% (*w*/*v*) PEI or 10% (*w*/*v*) DS.

The treatment with GA was performed according to Wang et al. [101], adding 0.4 mL of GA solution (25% in H_2_O) in 10 mL of immobilized enzyme solution (0.1 g/mL) prepared in 5 mM phosphate buffer at pH 8.0. The modification was carried out at room temperature under gentle agitation for 1 h. At the end, the suspensions were vacuum filtered, washed 10-fold with 20 volumes of distilled water, and stored at 4–6 °C.

For surface amination of the immobilized enzyme, 1 g of immobilized enzyme was added to 10 mL of 2 M EDA at pH 4.75. Then, solid ECD was added to reach a concentration of 10 mM. The amination was carried out at room temperature for 2 h. Under these conditions, 100% modification of all the exposed carboxylic groups was achieved [74]. At the end, the suspensions were vacuum filtered, washed 10-fold with 20 volumes of dissolved water, and stored at 4–6 °C.

The enzyme surface modification with TNBS followed the methodology of Snyder and Sobocinski [76]. A total of 58.8 μL of TNBS (1 mM final concentration) was added to 10 mL of solution containing the immobilized enzyme (0.1 g/mL) prepared in 5 mM sodium phosphate at pH 8.0. The modification was carried out at room temperature under gentle agitation for 3 h. At the end, the suspensions were vacuum filtered, washed 10-fold with 20 volumes of distilled water, and stored at 4–6 °C.

The modification with PEI or DS followed the methodology of Arana-Peña et al. [99] and Virgen-Ortíz et al. [102], respectively. An amount of 1 g of immobilized enzymes was treated with 10 mL of a solution 10% (*w/v*) of PEI or DS at pH 7.0. The modification was carried out at room temperature under gentle agitation for 18 h. Afterward, the biocatalysts were vacuum filtered, washed 10-fold with 20 volumes of distilled water, and stored at 4–6 °C.

#### 3.2.3. Modification of Immobilized Enzyme with Metallic Salt/Phosphate

The immobilized enzymes were modified with metallic salt/phosphate following the methodology described by Guimarães et al. [32]. A total of 400 μL of the metallic salt solution was added to 5 mL of the immobilized enzymes solution (0.1 g/mL) prepared in 10 mM sodium phosphate/125 mM NaCl at pH 7.4. The enzyme treatment was conducted at room temperature under gentle stirring for 5 h. Afterward, the biocatalysts were vacuum filtered, washed 10-fold with 20 volumes of distilled water, and stored at 4–6 °C.

#### 3.2.4. Thermal Inactivation of Different Lipase Biocatalysts

In a standard experiment, 1 g of immobilized biocatalyst was suspended in 10 mL of 10 mM Tris-HCl at pH 7.0 and incubated at 68 °C or 75 °C. Periodically, samples of 50 μL of the inactivation suspensions were collected to determine their residual activities. Residual activities were defined as the current activity divided by the initial one in percentage. The experiments were performed employing *p*-NPB as a substrate.

#### 3.2.5. Determination of the Biocatalysts Activities versus Different Substrates

One unit of activity (U) was defined as the amount of enzyme that hydrolyzes one µmol of substrate per minute under the described conditions. Considering the strong tendency of lipases, and specifically TLL, to form lipase–lipase dimers by involving the open forms of two lipase molecules, the use of free enzymes could drive complex results, so we preferred to focus on the comparison of the different immobilized enzymes [103,104,105,106,107,108,109]. Octyl agarose provided the monomeric and open form of the lipases, preventing this kind of problem [27,68].

##### Hydrolysis of *p*-NPB

A total of 50 μL of 50 mM *p*-NPB prepared in acetonitrile was added to 2.5 mL of 25 mM sodium phosphate at pH 7.0, and the reaction was started by adding 50 μL of soluble or immobilized enzyme sample to this mixture. The reaction was conducted using a thermostatization system at 25 °C under magnetic stirring for 1.5 min. The *p*-nitrophenol released into the medium was monitored by spectrophotometry at 348 nm (isosbestic point) to determine the hydrolytic activity (ε = 5150 M^−1^ cm^−1^) [110].

##### Hydrolysis of Triacetin

A total of 50 mg of immobilized enzyme was added to 3 mL of 50 mM of triacetin prepared in 50 mM of sodium phosphate at pH 7.0. The reaction was carried out at room temperature under gentle stirring. The quantification of hydrolysis degree was determined by the release of 1,2 and 1,3 diacetin (under these conditions, the 1,2 diacetin produced undergoes acyl migration giving 1,3 diacetin) in the reaction medium [111]. A Waters 486 chromatograph (Waters, Millford, MA, USA) presenting a Kromasil C18 column (15 cm × 0.46 cm) and a UV/VIS detector (set to 230 nm) was employed in the analyses to determine the degree of conversion (two points over 5% and under 25%, to ensure linearity and minimize experimental error caused by the initial acid content of the samples) and enzymatic activity. The mobile phase was composed of 85% (*v*/*v*) water and 15% (*v*/*v*) acetonitrile with a flow rate of 1 mL/min. The retention times were 4 min for 1,2 and 1,3 diacetins (under these conditions, they eluted at the same retention time) and 18 min for triacetin [99].

##### Hydrolysis of *R*- or *S*-Methyl Mandelate

A total of 50 mg of immobilized lipase was added to 3 mL of 50 mM *R*- or *S*-methyl mandelate in 50 mM sodium phosphate solution at pH 7.0. The reaction was carried out at room temperature under gentle stirring. The quantification of hydrolysis was determined by the release of mandelic acid in the reaction medium. A Waters 486 chromatograph (Waters, Millford, USA) presenting a Kromasil C18 column (15 cm × 0.46 cm) and a UV/VIS detector (set to 230 nm) was employed in the analyses to determine the degree of conversion (two points over 5% and under 25%, to ensure linearity and minimize experimental error caused by the initial acid content of the samples) and enzymatic activity [112]. The mobile phase was 10 mM ammonium acetate and acetonitrile (65–35% (*v*/*v*)) at pH 2.8 with a flow rate of 1 mL/min. The retention times were 2.5 min for mandelic acid and 4.2 min for the *R*- or *S*-methyl mandelate [113]. The activities ratio was defined as the activity versus the *R*-isomer/activity versus the *S*-isomer.

## 4. Conclusions

This paper shows, using two enzymes, how the previous physicochemical modification of immobilized lipases strongly affects the effects of mineralization on their catalytic properties. TLL was found to be more tunable than CALB, perhaps because of the smaller lid that CALB exhibits [114]; in any case, this enzyme usually is less tunable than other lipases (e.g., by the immobilization conditions) [115].

The effect of the same metallization may be positive for the biocatalyst activity versus one substrate and negative for the activity versus another substrate, and this effect depends on the enzyme and on their previous physicochemical modification. Similarly, the stability of the enzymes is altered by both physicochemical modification and mineralization, and these effects are not directly translated from one enzyme or biocatalyst to another. The mineralization of chemically or physically modified immobilized enzymes is a potent tool to improve enzyme features, but the effects cannot be predicted at this stage and must be empirically analyzed.

Investigating the actual causes of these effects on enzyme features would be a very interesting target. However, considering that the metal phosphate also forms crystals inside the supports without enzymes [32], the analysis may be hard. It could be highly interesting to investigate the different structural changes that the enzyme can adopt due to the different chemical and physical modifications, as this can help to design biocatalysts bearing the different activities and stabilities [112]. Unfortunately, although the potential of spectroscopic physico-chemical and biophysical techniques to advance in the structural analysis of immobilized enzymes and understand the structure–function relationship of the enzymes is advancing very rapidly [116,117,118,119,120,121,122,123,124,125], nowadays, they have many limits [116,117]. Therefore, these techniques are still far from providing a clear and unique vision that can explain the functional features of an immobilized enzyme, even though this is of great interest to improve the understanding of the phenomena that determine the final properties of the immobilized enzyme and may open new opportunities in the development of a more controllable and efficient immobilization process.

## Figures and Tables

**Table 1:** Specific activity of different biocatalysts with 1 mM *p*-NPB (pH 7 and 25 °C), 50 mM triacetin (pH 7 and 25 °C), and 50 mM *R*- or *S*-methyl mandelate (pH 7 and 25 °C). Experiments were conducted as described in Methods.

Biocatalysts	Activity (U/g)			
	*p*-NPB	Triacetin	*R*-Methyl Mandelate	*S*-Methyl Mandelate
Octyl-TLL	1416 ± 51	601 ± 6	1.2 ± 0.1	3.2 ± 0.2
Octyl-TLL-GA	1350 ± 91	48 ± 2	0.8 ± 0.1	2.4 ± 0.1
Octyl-TLL-Amin	1675 ± 49	88 ± 4	0.6 ± 0.1	3.2 ± 0.2
Octyl-TLL-TNBS	1764 ± 82	60 ± 3	0.9 ± 0.1	3.1 ± 0.2
Octyl-TLL-PEI	1363 ± 61	90 ± 4	9.8 ± 0.5	7.7 ± 0.4
Octyl-TLL-DS	985 ± 52	28 ± 1	7.5 ± 0.4	6.5 ± 0.2

**Table 2:** Specific activity of different TLL biocatalysts with 1 mM *p*-NPB (pH 7 and 25 °C), 50 mM triacetin (pH 7 and 25 °C), and 50 mM *R*- or *S*-methyl mandelate (pH 7 and 25 °C). Experiments were conducted as described in Methods.

Biocatalysts	Activity (U/g)			
	*p*-NPB	Triacetin	*R*-Methyl Mandelate	*S*-Methyl Mandelate
Octyl-TLL	1416 ± 51	61 ± 6	1.2 ± 0.1	3.2 ± 0.2
Octyl-TLL-ZnP *	1040 ± 64	88 ± 6	0.9 ± 0.1	3.0 ± 0.1
Octyl-TLL-CoP	1207 ± 72	65 ± 5	1.1 ± 0.1	2.9 ± 0.2
Octyl-TLL-CuP	1144 ± 90	67 ± 6	0.8 ± 0.1	2.7 ± 0.1
Octyl-TLL-NiP	1371 ± 87	53 ± 3	1.5 ± 0.1	3.0 ± 0.2
Octyl-TLL-MgP	1665 ± 41	66 ± 3	1.5 ± 0.1	2.4 ± 0.1

* ZnP, CuP, CoP, NiP, MgP correspond to the metallic salt/sodium phosphate.

**Table 3:** Specific activity of different immobilized and chemically modified TLL biocatalysts after mineralization. The hydrolytic activity was measured using 1 mM *p*-NPB (pH 7 and 25 °C), 50 mM triacetin (pH 7 and 25 °C), and 50 mM *R*- or *S*-methyl mandelate (pH 7 and 25 °C). Experiments were conducted as described in Methods.

Line	Biocatalysts	Activity (U/g)			
1		*p*-NPB	Triacetin	*R*-Methyl Mandelate	*S*-Methyl Mandelate
2	Octyl-TLL-GA	1350 ± 91	48 ± 2	0.8 ± 0.1	2.4 ± 0.1
3	Octyl-TLL-GA-ZnP	1408 ± 84	50 ± 2	0.7 ± 0.1	3.0 ± 0.1
4	Octyl-TLL-GA-CoP	1133 ± 61	35 ± 2	0.2 ± 0.1	2.0 ± 0.1
5	Octyl-TLL-GA-CuP	1142 ± 42	38 ± 2	0.6 ± 0.1	2.1 ± 0.1
6	Octyl-TLL-GA-NiP	1308 ± 54	48 ± 2	0.6 ± 0.1	2.2 ± 0.1
7	Octyl-TLL-GA-MgP	1404 ± 120	46 ± 2	0.7 ± 0.1	2.4 ± 0.1
8	Octyl-TLL-Amin	1675 ± 49	88 ± 4	0.6 ± 0.1	3.2 ± 0.2
9	Octyl-TLL-Amin-ZnP	1422 ± 208	78 ± 4	0.6 ± 0.1	3.1 ± 0.2
10	Octyl-TLL-Amin-CoP	1647 ± 87	53 ± 3	0.1 ± 0.1	2.2 ± 0.1
11	Octyl-TLL-Amin-CuP	1618 ± 36	79 ± 4	0.9 ± 0.4	3.0 ± 0.2
12	Octyl-TLL-Amin-NiP	1863 ± 107	63 ± 3	0.6 ± 0.1	2.8 ± 0.1
13	Octyl-TLL-Amin-MgP	1979 ± 90	79 ± 4	0.8 ± 0.1	2.9 ± 0.1
14	Octyl-TLL-TNBS	1763 ± 82	60 ± 3	0.9 ± 0.1	3.1 ± 0.2
15	Octyl-TLL-TNBS-ZnP	1836 ± 97	47 ± 2.	0.9 ± 0.1	2.7 ± 0.1
16	Octyl-TLL-TNBS-CoP	1591 ± 109	46 ± 2	0.4 ± 0.1	2.4 ± 0.1
17	Octyl-TLL-TNBS-CuP	1501 ± 76	45 ± 2	1.0 ± 0. 14	3.0 ± 0.2
18	Octyl-TLL-TNBS-NiP	1592 ± 128	44 ± 2	1.4 ± 0.1	3.1 ± 0.2
19	Octyl-TLL-TNBS-MgP	1544 ± 60	46 ± 2	1.1 ± 0.1	3.0 ± 0.2

ZnP, CuP, CoP, NiP, MgP, ZnP, CuP, CoP, NiP, MgP correspond to the metallic salt/sodium phosphate.

**Table 4:** Specific activity of different immobilized and physically modified biocatalysts after solid-phase mineralization. The hydrolytic activity was measured using 1 mM *p*-NPB (pH 7 and 25 °C), 50 mM triacetin (pH 7 and 25 °C), and 50 mM *R*- or *S*-methyl mandelate (pH 7 and 25 °C). Experiments were conducted as described in Methods.

Line	Biocatalysts	Activity (U/g)			
1		*p*-NPB	Triacetin	*R*-Methyl Mandelate	*S*-Methyl Mandelate
2	Octyl-TLL-PEI	1363 ± 61	90 ± 4	9.8 ± 0.5	7.7 ± 0.4
3	Octyl-TLL-PEI-ZnP	1479 ± 20	106 ± 5	8.2 ± 0.4	8.2 ± 0.5
4	Octyl-TLL-PEI-CoP	1442 ± 31	108 ± 6	7.7 ± 0.4	8.1 ± 0.3
5	Octyl-TLL-PEI-CuP	1351 ± 80	120 ± 6	8.7 ± 0.4	8.0 ± 0.2
6	Octyl-TLL-PEI-NiP	1309 ± 72	94 ± 4	8.5 ± 0.5	7.9 ± 0.3
7	Octyl-TLL-PEI-MgP	1323 ± 50	80 ± 5	8.2 ± 0.4	7.7 ± 0.3
8	Octyl-TLL-DS	985 ± 52	28 ± 1	7.5 ± 0.4	6.5 ± 0.2
9	Octyl-TLL-DS-ZnP	1041 ± 31	37 ± 2	9.9 ± 0.4	7.3 ± 0.4
10	Octyl-TLL-DS-CoP	1084 ± 46	27 ± 1	8.1 ± 0.3	6.1 ± 0.2
11	Octyl-TLL-DS-CuP	898 ± 54	30 ± 2	8.9 ± 0.4	7.2 ± 0.4
12	Octyl-TLL-DS-NiP	1041 ± 40	28 ± 1	8.5 ± 0.5	7.2 ± 0.3
13	Octyl-TLL-DS-MgP	951 ± 23	31 ± 1	8.9 ± 0.4	7.4 ± 0.4

ZnP, CuP, CoP, NiP, MgP correspond to the metallic salt/sodium phosphate.

**Table 5:** Specific activity of different CALB biocatalysts with 1 mM *p*-NPB (pH 7 and 25 °C), 50 mM triacetin (pH 7 and 25 °C), and 50 mM *R*- or *S*-methyl mandelate (pH 7 and 25 °C). Experiments were conducted as described in Methods.

Biocatalysts	Activity (U/g)			
	*p*-NPB	Triacetin	*R*-Methyl Mandelate	*S*-Methyl Mandelate
Octyl-CALB	1151 ± 62	132 ± 6	19.5 ± 0.9	39.0 ± 1.9
Octyl-CALB-GA	1325 ± 23	94 ± 5	16.8 ± 0.9	28.0 ± 1.4
Octyl-CALB-Amin	1312 ± 36	77 ± 3	26.5 ± 1.3	36.3 ± 1.9
Octyl-CALB-TNBS	1336 ± 36	89 ± 7	18.3 ± 0.8	41.1 ± 2.0
Octyl-CALB-PEI	1518 ± 91	143 ± 9	26.1 ± 1.5	33.3 ± 1.7
Octyl-CALB-DS	1294 ± 85	121 ± 4	20.0 ± 0.8	43.2 ± 2.2

**Table 6:** Specific activity of different CALB biocatalysts with 1 mM *p*-NPB (pH 7 and 25 °C), 50 mM triacetin (pH 7 and 25 °C), and 50 mM *R*- or *S*-methyl mandelate (pH 7 and 25 °C). Experiments were conducted as described in Methods.

Biocatalysts	Activity (U/g)			
	*p*-NPB	Triacetin	*R*-Methyl Mandelate	*S*-Methyl Mandelate
Octyl-CALB	1151 ± 62	132 ± 6	19.5 ± 0.9	39.0 ± 1.9
Octyl-CALB-ZnP	1172 ± 43	194 ± 7	25.6 ± 1.3	49.5 ± 2.9
Octyl-CALB-CoP	967 ± 52	220 ± 13	27.7 ± 1.4	50.2 ± 2.2
Octyl-CALB-CuP	998 ± 73	270 ± 14	26.8 ± 1.3	50.1 ± 2.9
Octyl-CALB-NiP	1218 ± 78	222 ± 13	20.4 ± 1.0	43.5 ± 2.1
Octyl-CALB-MgP	1141 ± 49	82 ± 5	17.5 ± 0.9	29.5 ± 1.6

ZnP, CuP, CoP, NiP, MgP correspond to the metallic salt/sodium phosphate.

**Table 7:** Specific activity of different immobilized and chemically modified CALB biocatalysts after solid-phase mineralization. The hydrolytic activity was measured using 1 mM *p*-NPB (pH 7 and 25 °C), 50 mM triacetin (pH 7 and 25 °C), and 50 mM *R*- or *S*-methyl mandelate (pH 7 and 25 °C). Experiments were conducted as described in Methods.

Line	Biocatalysts	Activity (U/g)			
1		*p*-NPB	Triacetin	*R*-Methyl Mandelate	*S*-Methyl Mandelate
2	Octyl-CALB-GA	1324 ± 23	94 ± 5	16.8 ± 0.9	28.0 ± 1.4
3	Octyl-CALB-GA-ZnP	1195 ± 23	92 ± 5	9.7 ± 0.5	25.0 ± 1.9
4	Octyl-CALB-GA-CoP	1324 ± 39	85 ± 4	15.5 ± 0.8	26.6 ± 1.3
5	Octyl-CALB-GA-CuP	1195 ± 29	82 ± 4	14.1 ± 0.7	31.4 ± 1.4
6	Octyl-CALB-GA-NiP	1263 ± 44	78 ± 2	18. 9 ± 0.8	27.8 ± 1.2
7	Octyl-CALB-GA-MgP	1264 ± 56	121 ± 6	23.8 ± 1.0	29.2 ± 1.6
8	Octyl-CALB-Amin	1312 ± 36	77 ± 3	26.5 ± 1.3	36.3 ± 1.9
9	Octyl-CALB-Amin-ZnP	1266 ± 21	112 ± 7	23.6 ± 1.4	27.9 ± 1.3
10	Octyl-CALB-Amin-CoP	1225 ± 69	122 ± 6	25.3 ± 1.1	27.0 ± 1.0
11	Octyl-CALB-Amin-CuP	1341 ± 63	97 ± 6	24.3 ± 0.9	27.6 ± 1.2
12	Octyl-CALB-Amin-NiP	1369 ± 102	116 ± 6	24.7 ± 1.4	37.0 ± 1.9
13	Octyl-CALB-Amin-MgP	1264 ± 56	121 ± 6	23.8 ± 1.0	29.2 ± 1.6
14	Octyl-CALB-TNBS	1336 ± 36	89 ± 7	18.3 ± 0.8	41.1 ± 2.0
15	Octyl-CALB-TNBS-ZnP	1266 ± 70	139 ± 7	16.7 ± 0.8	39.1 ± 2.0
16	Octyl-CALB-TNBS-CoP	1263 ± 81	140 ± 8	17.4 ± 0.7	37.5 ± 1.9
17	Octyl-CALB-TNBS-CuP	1128 ± 58	169 ± 8	19.3 ± 0.9	43.3 ± 2.1
18	Octyl-CALB-TNBS-NiP	1214 ± 119	153 ± 7	20.0 ± 0.8	38.2 ± 1.9
19	Octyl-CALB-TNBS-MgP	1170 ± 34.9	145 ± 9	18.6 ± 1.2	39.8 ± 2.0

ZnP, CuP, CoP, NiP, MgP correspond to the metallic salt/sodium phosphate.

**Table 8:** Specific activity of different immobilized and physically modified CALB biocatalysts after solid-phase mineralization. The hydrolytic activity was measured using 1 mM *p*-NPB (pH 7 and 25 °C), 50 mM triacetin (pH 7 and 25 °C), and 50 mM *R*- or *S*-methyl mandelate (pH 7 and 25 °C). Experiments were conducted as described in Methods.

Line	Biocatalysts	Activity (U/g)			
1		*p*-NPB	Triacetin	*R*-Methyl Mandelate	*S*-Methyl Mandelate
2	Octyl-CALB-PEI	1518 ± 91	143 ± 9	26.1 ± 1.5	33.3 ± 1.7
3	Octyl-CALB-PEI-ZnP	1769 ± 30	181 ± 7	26.9 ± 1.4	38.2 ± 2.0
4	Octyl-CALB-PEI-CoP	1638 ± 33	201 ± 11	26.3 ± 1.4	40.8 ± 2.1
5	Octyl-CALB-PEI-CuP	1656 ± 48	219 ± 14	17.2 ± 0.9	45.9 ± 2.9
6	Octyl-CALB-PEI-NiP	1489 ± 93	200 ± 11	12.5 ± 0.7	47.8 ± 2.3
7	Octyl-CALB-PEI-MgP	1468 ± 89	207 ± 13	19.1 ± 1.0	48.5 ± 2.6
8	Octyl-CALB-DS	1294 ± 85	121 ± 4	20.0 ± 0.8	43.2 ± 2.2
9	Octyl-CALB-DS-ZnP	1251 ± 51	132 ± 8	23.6 ± 1.1	43.2 ± 1.9
10	Octyl-CALB-DS-CoP	1248 ± 57	144 ± 8	21.4 ± 1.0	44.8 ± 2.6
11	Octyl-CALB-DS-CuP	1188 ± 101	182 ± 10	20.9 ± 0.8	46.6 ± 2.5
12	Octyl-CALB-DS-NiP	1232 ± 31	159 ± 9	19.3 ± 0.6	41.0 ± 1.9
13	Octyl-CALB-DS-MgP	1308 ± 27	179 ± 10	21.5 ± 0.9	44.3 ± 1.4

ZnP, CuP, CoP, NiP, MgP correspond to the metallic salt/sodium phosphate.

## Data Availability

Not applicable.

## Supplementary Materials

The supporting information can be downloaded at: https://www.mdpi.com/article/10.3390/ijms232112808/s1.
