# Supplementary material for: Tuning Immobilized Enzyme Features by Combining Solid-Phase Physicochemical Modification and Mineralization
Source: Int J Mol Sci. 2022 Oct 24;23(21):12808. doi: 10.3390/ijms232112808 (PMC9658946; doi:10.3390/ijms232112808)
Supplement: Supplementary file 1 [file ijms-23-12808-s001.zip › ijms-1947719-supplementary.pdf]

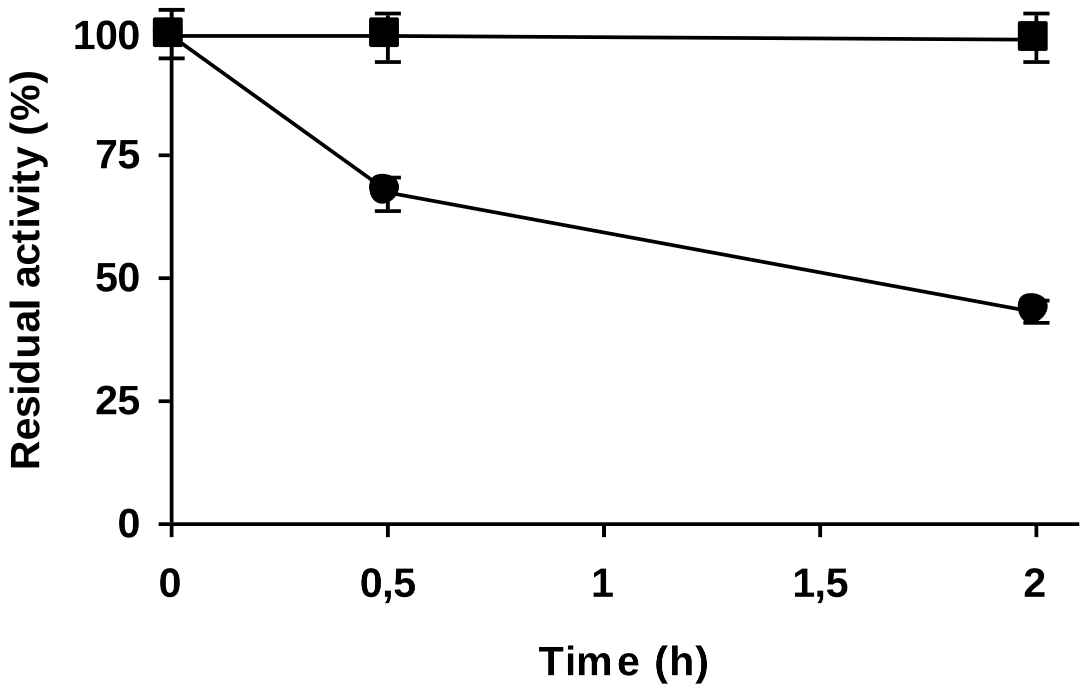

Figure S1. Immobilization course of TLL over octyl agarose using enzyme loading of 20 mg/g. The immobilization was performed in 5 mM sodium phosphate at 25 °C and pH 7.0. Squares: reference and circles: supernatant. Other specifications are described in Methods.

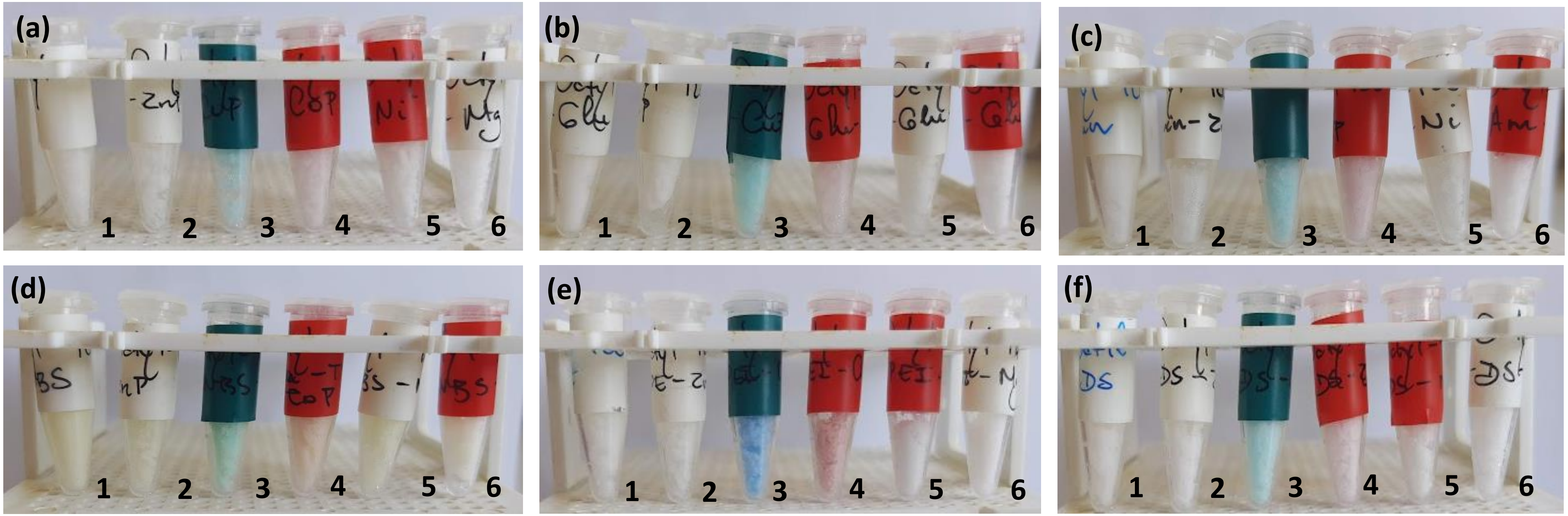

Figure S2. **(a)** Octyl-TLL biocatalysts modified with **(b)** 1% glutaraldehyde, **(c)** amination using 2 M ethylenediamine, **(d)** 1 mM picrylsulfonic acid, **(e)** 10% polyethylenimine, and **(f)** 10% dextran sulfate **(1)** untreated and **(2-6)** treated with **(2)** $ZnCl_2$/ sodium phosphate, **(3)** $CuCl_2$/sodium phosphate, **(4)** $CoCl_2$/sodium phosphate, **(5)** $NiCl_2$/sodium phosphate, and **(6)** $MgCl_2$/sodium phosphate. Other specifications are described in Methods.

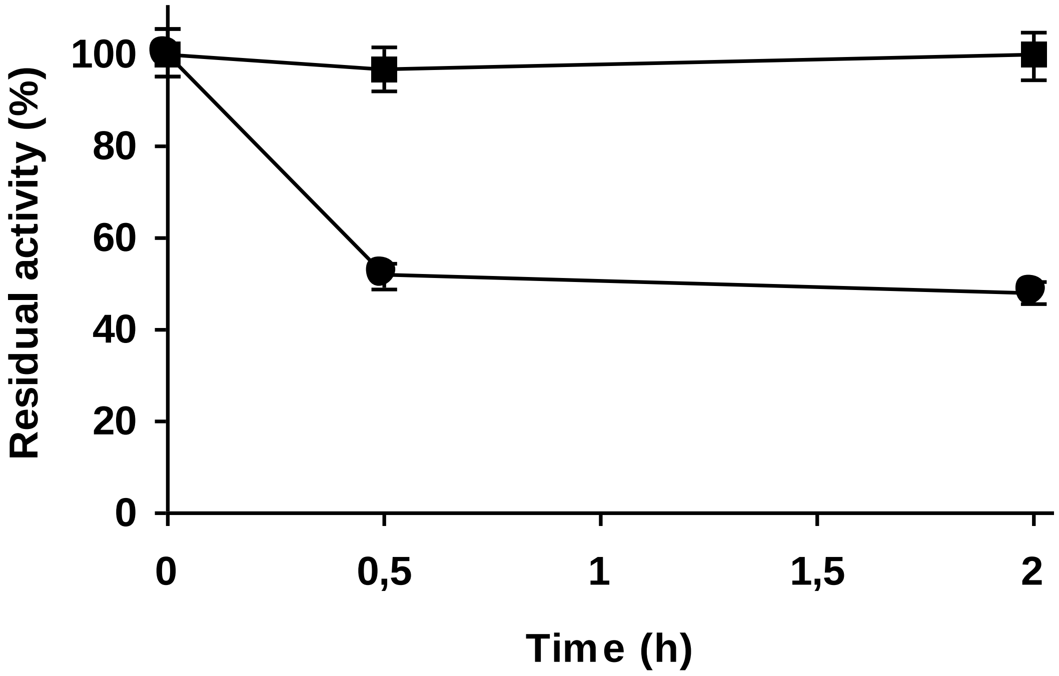

Figure S3. Immobilization course of CALB over octyl agarose using enzyme loading of 20 mg/g. The immobilization was performed in 5 mM sodium phosphate at 25 °C and pH 7.0. Squares: reference and circles: supernatant. Other specifications are described in Methods.

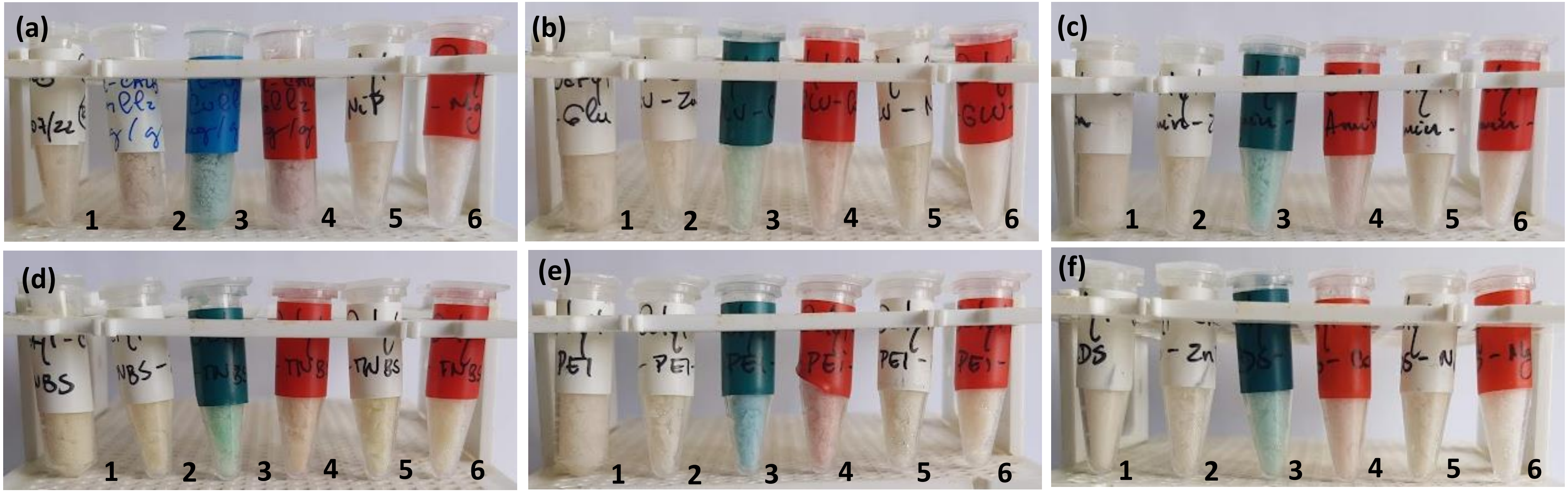

Figure S4. **(a)** Octyl-CALB biocatalysts modified with **(b)** 1% glutaraldehyde, **(c)** amination using 2 M ethylenediamine, **(d)** 1 mM picrylsulfonic acid, **(e)** 10% polyethylenimine, and **(f)** 10% dextran sulfate **(1)** untreated and **(2-6)** treated with **(2)** $ZnCl_2$/sodium phosphate, **(3)** $CuCl_2$/sodium phosphate, **(4)** $CoCl_2$/sodium phosphate, **(5)** $NiCl_2$/sodium phosphate, and **(6)** $MgCl_2$/sodium phosphate. Other specifications are described in Methods.
